# Protective Effects of MDG-1, a Polysaccharide from *Ophiopogon japonicus* on Diabetic Nephropathy in Diabetic KKA^y^ Mice

**DOI:** 10.3390/ijms160922473

**Published:** 2015-09-17

**Authors:** Yuan Wang, Lin-Lin Shi, Ling-Yi Wang, Jin-Wen Xu, Yi Feng

**Affiliations:** 1 Engineering Research Center of Modern Preparation Technology of TCM, Shanghai University of Traditional Chinese Medicine, Shanghai 201203, China; E-Mails: amoness@163.com (Y.W.); newhelenhappy@163.com (L.-L.S.); shilinmochen@126.com (L.-Y.W.); 2 Murad Research Institute for Modernized Chinese Medicine, Shanghai University of Traditional Chinese Medicine, Shanghai 201203, China

**Keywords:** diabetic nephropathy, *Ophiopogon japonicus*, polysaccharide

## Abstract

*Ophiopogon japonicus* is a traditional Chinese medicine that might be effective for treating type 2 diabetes. Recent research confirmed that MDG-1, a polysaccharide from *O. japonicas*, activates the PI3K/Akt signaling pathway and improves insulin sensitivity in a diabetic KKA^y^ mouse model, but little is known about its effects on diabetic nephropathy. In this study, KKA^y^ mice were orally administered distilled water (control group), MDG-1, or rosiglitazone for 12 weeks. Blood glucose levels were tested every two weeks for the fed mice. At 6 and 12 weeks, blood samples were collected for biochemical examination. At the end of the experiment, all kidney tissues were collected for histological examination and western blot analysis. Results show that MDG-1 (300 mg/kg) significantly decreased the levels of blood glucose, triglycerides, blood urine nitrogen and albumin, and significantly inhibited the expression of transforming growth factor-beta 1 and connective tissue growth factor. Moreover, MDG-1 could alleviate glomerular mesangial expansion and tubulointerstitial fibrosis in the diabetic mice, as confirmed by histopathological examination. These data indicated that MDG-1 ameliorates renal disease in diabetic mice by reducing hyperglycemia, hyperinsulinemia, and hyperlipidemia, and by inhibiting intracellular signaling pathways.

## 1. Introduction

Diabetes mellitus is a chronic metabolic disease caused mainly by insulin deficiency or insulin resistance [1]. Both type 1 and type 2 diabetes are characterized by chronic hyperglycemia, with glucose contributing to diabetic complications such as retinopathy, neuropathy, nephropathy and cardiomyopathy [2]. Among these complications, diabetic nephropathy (DN) is considered the most common cause of end-stage renal disease in many developed countries, and is a major cause of global morbidity and mortality in patients with kidney diseases [3,4].

DN is characterized by a series of structure abnormalities in the diabetic kidney, including expansion of mesangial cells, accumulation of extracellular matrix (ECM) proteins, thickening of glomerular and tubular basement membranes, tubulointerstitial fibrosis, glomerulosclerosis, and renal endothelial dysfunction [5].

Although the underlying pathogenic mechanism of DN has not been determined, some growth factors have been suggested to contribute to its progression. Transforming growth factor-beta 1 (TGF-β_1_) is widely expressed in all cells, including the kidney, where it exerts proinflammatory and profibrotic effects, such as mediating ECM deposition, increasing the synthesis of matrix components and reducing their degradation [6]. Induction of TGF-β_1_ overexpression in glomeruli and tubules is accompanied by a sclerosing reaction in renal diseases that have common permselective defects of the glomerular barrier, irrespective of their etiology [7]. Connective tissue growth factor (CTGF) is a downstream target of TGF-β_1_ and is a potent inducer of ECM in the fibrotic process [8]. To prevent the development and progression of DN, effective therapies directed toward key molecular targets are required [9].

To date, only a few effective drugs against DN are available, such as losartan, irbesartan, rosiglitazone (RGZ) or captopril. Unfortunately, most of them have been reported to have only limited beneficial effect during long-term treatment [10,11]. However, many traditional Chinese medicines have been used in clinical practice and show promising results in the therapy of diabetes and its complications, according to traditional medical theory.

**Figure 1:**
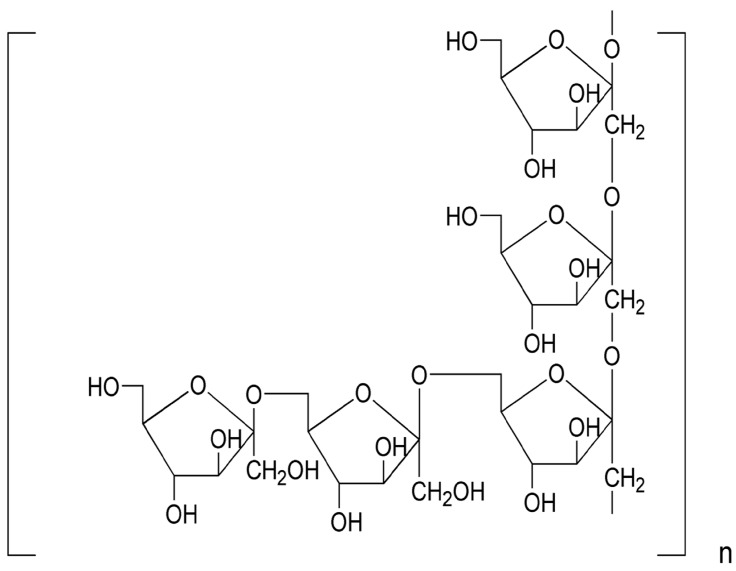
The repeating unit structure of MDG-1 from *O. japonicus.*

*Ophiopogon japonicus* is widely distributed in Southeast Asia, and its radix is the primary medical portion that has been used as a traditional Chinese medicine to treat diabetes and cardiovascular diseases for thousands of years [12]. Our previous reports demonstrated that MDG-1, a polysaccharide extracted from the roots of *O. japonicus*, is a water-soluble β-d-fructan with an average molecular weight of 3400 Da, including a backbone composed of Fruf (2→1) and a branch of Fruf (2→6). Fruf (2→) per average 2.8 of main chain residues. MDG-1 also contains trace of α-d-GLc [13], which may be connected to its reducing terminal (Figure 1). Recent research has suggested that MDG-1 could reduce hyperglycemia, hyperinsulinemia and hyperlipidemia in the spontaneous model of type 2 diabetes in *ob*/*ob* mice or KKA^y^ mice [14,15,16]. The aim of this study was to evaluate the potential effect of MDG-1 against the DN, as well as the mechanisms underlying this potential protective activity in KKA^y^ mice.

## 2. Results and Discussion

DN is one of the major microvascular complications of diabetes and is a leading cause of end-stage renal disease [17]. Prevention of the initiation of nephropathy or retardation of the progression of glomerular sclerosis (GS) is the goal for the development of therapeutic agents for renal disease [18]. Despite the important progress made in the management of diseases using synthetic drugs, there has been renewed interest in the use of herbal products during recent years [19]. In our previous studies, we demonstrated the hypoglycemic and insulin-sensitizing effects of MDG-1 in diabetic mice [14,15]. In this study, we explored the beneficial effect of MDG-1 in the prevention of diabetic nephropathy.

### 2.1. Effects of MDG-1 on Fed Blood Glucose and Serum Insulin Levels

Hyperglycemia is considered the main cause of diabetic complications. The KKA^y^ mice, which were produced by transfection of the yellow obese gene (A^y^) into KK mice, are obese, diabetic mice that show hyperglycemia, hypertriglyceridemia, hyperinsulinemia and microalbuminuria spontaneously at 20 weeks of age, compared with C57BL/6J mice. In addition, the renal lesions in these mice closely resemble those in human diabetic nephropathy. In this study, we adopted the KKA^y^ mouse as the model of DN to investigate the renoprotective effect of MDG-1.

**Figure 2:**
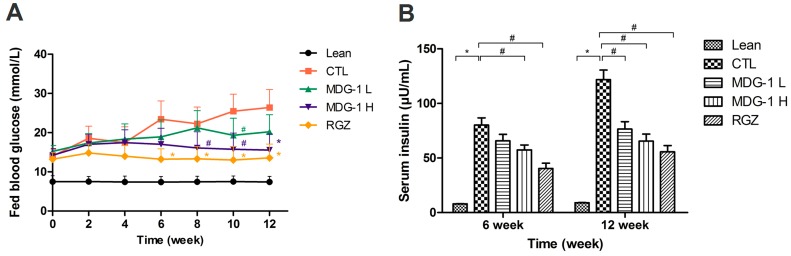
Effects of MDG-1 on fed blood glucose values (**A**) and serum insulin levels (**B**) in KKA^y^ mice. Data were expressed as mean values (±SD) from 10–13 mice. Lean, C57BL/6J mice; CTL, distilled water-treated KKA^y^ mice; MDG-1 L, KKA^y^ mice treated with 150mg/kg MDG-1; MDG-1 H, KKA^y^ mice treated with 300mg/kg MDG-1; RGZ, rosiglitazone-treated KKA^y^ mice. Significance: ^#^ *p* < 0.05, * *p* < 0.01 *vs*. control group.

The KKA^y^ mice were treated with 150 mg/kg MDG-1, 300 mg/kg MDG-1, 2 mg/kg RGZ or distilled water for 12 weeks; no obvious behavioral changes were noted in the MDG-1-treated groups. After the 12-week treatment period, a significant decrease in fed blood glucose levels was observed in the 300 mg/kg MDG-1 treated group. A dose-dependent reduction in fed blood glucose levels was observed after MDG-1 treatment (Figure 2A). During the 12-week treatment period, the fed blood glucose levels of the two MDG-1- and RGZ-treated groups decreased by 23.5%, 41.2% and 48.7%, respectively (mean decrease rate, ∑[(control group blood glucose value − treatment group blood glucose value)/control group blood glucose value × 100%]/*n*), compared with the control group (*p* < 0.05). The hypoglycemic effect of MDG-1 on the fed blood glucose levels at a dose of 300 mg/kg was similar to that of RGZ at a dose of 2 mg/kg in the diabetic KKA^y^ mice.

KKA^y^ mice are also characterized by advanced hyperinsulinemia. To elucidate the mechanism of MDG-1 action, the effect of its administration on serum insulin levels was investigated at the midpoint and at the end of the study. The serum insulin levels of fasting mice were significantly higher than those of the lean mice (*p* < 0.01) (Figure 2B). Treatment with 300 mg/kg MDG-1 for 6 weeks decreased the serum insulin levels significantly, whereas no obvious reduction was observed in the lower dosage MDG-1 treatment group. However, by the end of the study period, the down-regulating effect of MDG-1 on serum insulin levels was maintained and became more evident. Compared with the control group, both MDG-1 dosage groups showed an obvious ameliorative effect. The percentage reduction of average fasting serum insulin by 300 mg/kg MDG-1 reached 46.1% at 12 weeks, similar to that of the RGZ group (54.3%). These results suggest that MDG-1 exerts a hypoglycemic effect in diabetic KKA^y^ mice.

### 2.2. Effects of MDG-1 on Body Weight Gain, Lipid Disorder, and Renal Parameters

Body weight, kidney index, triglycerides, and the renal parameters of all treatment groups are summarized in Table 1). The body weight of the KKA^y^ mice was significantly lower than that of the C57BL/6J mice (*p* < 0.01). MDG-1 treatment ameliorated the body weight gain and kidney index in the KKA^y^ mice, even though this difference was not statistically significant, the body weight of MDG-1-treated groups were decreased compared to the control group. After the 12 weeks of treatment, the serum levels of TG in the 300 mg/kg MDG-1- and RGZ-treated KKA^y^ mice were significantly lower than those in the vehicle-treated KKA^y^ group. In addition, the 150 mg/kg MDG-1-treated group showed a similar attenuated effect on those serum parameters; however, the reduction was lower, except for the levels of TG.

The percentages of the decreased levels of TG and body weight in the treatment groups showed the potent lipid-lowering efficacy of MDG-1 in KKA^y^ mice. Furthermore, a number of recent studies have proven that improved metabolic control that achieves near-normoglycemia can significantly decrease the development and progression of DN [20,21,22]. We concluded that MDG-1 might have an anti-hyperglycemic and anti-hyperlipidemic effect that is correlated with a decrease in body weight.

Furthermore, DN is also characterized by increased urinary proteins and loss of renal function [23]. Microalbuminuria is a functional parameter in the early stages of DN. In fact, albuminuria in diabetes is considered having both hemodynamic (glomerular capillary hypertension and hyperfiltration) and structural/cellular causes (changes in GBM mesangial cell matrix, and podocyte function) [2,24]. We observed an increased concentration of the serum albumin in our KKA^y^ mice. Additionally, the serum BUN level and the kidney index, which are generally considered markers of renal function, were higher in the control mice than in the lean mice, implying the presence of diabetic kidney disease with renal hyperfiltration. The MDG-1-treated groups showed significant improvements in renal function, as indicated by serum BUN and albumin levels.

**Table 1:** Body weight, kidney index, and levels of four different serum blood parameters in 4 different treatment groups of KKA^y^ mice and the lean C57BL/6J mice.

Groups	Body Weight		Kidney Index		TC		TG		BUN		Albumin	
	(g)	%	(mg/g)	%	mmol/L	%	mmol/L	%	mg/dL	%	g/dL	%
Lean	32.12 ± 1.89 *	66	8.71 ± 0.31 ^#^	125	2.65 ± 0.32 *	50	0.92 ± 0.21 *	30	22.12 ± 4.15 *	63	2.44 ± 0.21 ^#^	69
Control	48.92 ± 2.09	100	11.38 ± 0.43	100	5.32 ± 0.38	100	3.11 ± 0.41	100	35.37 ± 6.23	100	3.52 ± 0.45	100
MDG-1 L	46.32 ± 2.34	95	10.12 ± 0.41	95	4.41 ± 0.43	83	2.34 ± 0.32 ^#^	75	31.39 ± 7.54	89	2.98 ± 0.35	85
MDG-1 H	44.14 ± 2.67	90	8.95 ± 0.37	94	4.15 ± 0.43 ^#^	78	1.68 ± 0.25 *	54	27.07 ± 6.73 ^#^	77	2.57 ± 0.25 ^#^	73
RGZ	43.79 ± 2.45	90	8.61 ± 0.36	92	3.87 ± 0.38 ^#^	73	1.65 ± 0.23 *	53	25.65 ± 7.02 ^#^	73	2.62 ± 0.31 ^#^	74

TC, total cholesterol; TG, triglycerides; BUN, blood urea nitrogen; RGZ, rosiglitazone; MDG-1 L and MDG-1 H groups were fed with MDG-1 150mg/kg.d and 300mg/kg.d body weight, respectively, for 12 weeks. Data are expressed as means (±SD). Significance: ^#^ *p* < 0.05, * *p* < 0.01, *vs*. control group.

### 2.3. Effects of MDG-1 on Renal Pathology

**Figure 3:**
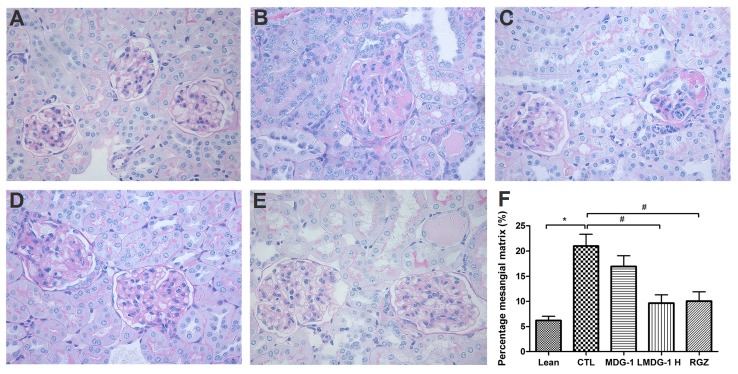
Effects of MDG-1 on renal pathology changes in KKA^y^ mice, as assessed by PAS staining (400× magnification). Representative microphotographs of the glomeruli are presented for the lean group (**A**); control group (**B**); MDG-1 L group (**C**); MDG-1 H group (**D**); and the rosiglitazone group (**E**); Picture (**F**) shows the quantitative assessment of the glomerular mesangial expansion in each group. Data are expressed as mean values (±SD). Significance: ^#^ *p* < 0.05, * *p* < 0.01 *vs*. control group.

**Figure 4:**
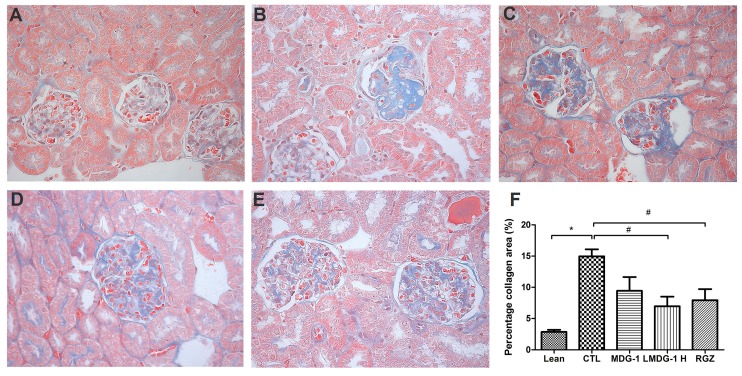
Effects of MDG-1 on renal pathology changes in KKA^y^ mice, as assessed by Masson’s trichrome staining (400× magnification). Representative microphotographs of the glomeruli are shown for the lean group (**A**); control group (**B**); MDG-1 L group (**C**); MDG-1 H group (**D**); and the RGZ group (**E**); Picture (**F**) shows the quantitative assessment of tubule-interstitial collagen area of the experimental mice in each group. Data are expressed the mean values (±SD). Significance: ^#^ *p* < 0.05, * *p* < 0.01 *vs*. control group.

PAS and Masson’s stains were used to examine the glomerular and tubular structures, respectively, to assess the protective effect of MDG-1 on DN. Under light microscopy, no obvious abnormalities in the glomerular and tubular structures of the lean mice kidneys were observed (Figure 3). However, diffuse mesangial matrix expansion, thickened basement membranes, and occluded capillaries were detected in the control mice. Both MDG-1 and RGZ treatment significantly reduced the percentage of mesangial matrix and showed a protective effect on renal tubule lesions. Interstitial and glomerular fibroses were further confirmed by Masson’s trichrome staining. MDG-1 treatment markedly attenuated the fibrotic process that was prominent in the control mice (Figure 4). These effects are interesting, because structural ECM alterations correlate with DN severity [25].

### 2.4. Expression on TGF-β_1_ and CTGF Proteins

Much attention has been paid to exploring the mechanisms related to the development of DN. Currently, several growth factors are thought to be involved in mediating the development of diabetic renal hypertrophy. Among them, the multifunctional cytokine TGF-β_1_ is up-regulated in diabetic kidneys [26]. Numerous studies indicate that hyperglycemia induces an increase in TGF-β_1_ expression at both the mRNA and protein levels in glomerular and tubular compartments of various models of experimental diabetes in rats and mice [27]. TGF-β_1_ stimulates the synthesis of key ECM molecules in diabetic glomeruli, especially fibronectin and type IV collagen. Fibronectin and collagen IV are both present in the normal mesangium, but are increased in DN, and increased collagen IV expression in the GBM contributes to its thickening in DN. CTGF, growth factors only recently discovered to have a role in DN, is a downstream mediator of TGF-β_1_ and a potent inducer of ECM in the fibrotic process [28]. Some researchers believe that TGF-β_1_/Smad signaling is largely responsible for CTGF expression. However, there is also evidence of a TGF-β-independent regulation of CTGF, which is related to a direct activation of its synthesis by hyperglycemia, advanced glycosylation end products and static pressure [29].

**Figure 5:**
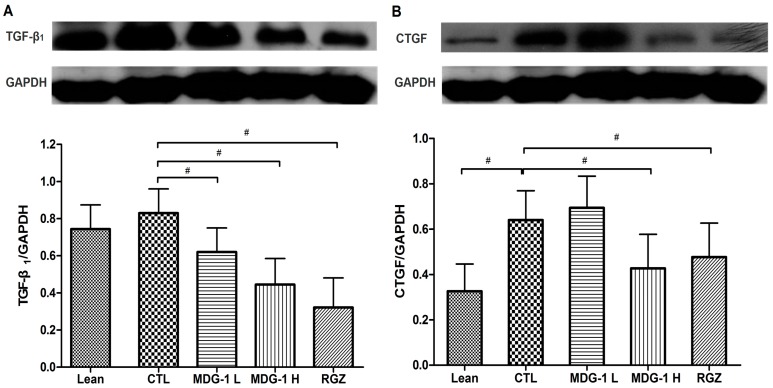
Effects of MDG-1 on TGF-β1 (**A**) and CTGF (**B**) in KKA^y^ mice kidneys, as assessed by western blotting. **Top** panel: Western blot; **bottom** panel: Histogram of band densities. Data are expressed as mean values (±SD). Significance: ^#^ *p* < 0.05 *vs*. control group.

Our western blot analysis showed that the expressions of TGF-β_1_ and CTGF were elevated in the control group, while MDG-1 treatment reversed these changes in diabetic kidneys, suggesting that both factors play a key role in the development of fibrosis in diabetic nephropathy. To be specific, the diabetic control mice showed a 2-fold higher TGF-β_1_ and CTGF protein expression than the lean mice and treatment with 300 mg/kg MDG-1 reduced the TGF-β_1_ and CTGF expression in KKA^y^ mice by 28% and 48%, respectively (*p* < 0.05) (Figure 5). These results suggested that MDG-1 improves the renal function via modulation of the TGF-β_1_ signaling pathway.

## 3. Experimental Section

### 3.1. Materials and Chemicals

MDG-1 was extracted from the tube root of *O. japonicus* (Cixi, Zhejiang Province, China) and purified as described previously [13]. The antibodies to CTGF (ab51704) and TGF-β_1_ (ab51704) were purchased from Abcam (Cambridge, UK). GAPDH (1105) was purchased from Kang Cheng (Beijing, China). As secondary antibodies, we used either peroxidase-conjugated goat anti-rabbit IgG or peroxidase-conjugated goat anti-mouse IgG from Santa Cruz Biotechnology (Santa Cruz, CA, USA). Insulin ELISA Kits (EZRMI-13K) were purchased from Millipore Corporation (Bedford, MA, USA).

### 3.2. Animals

Fifty male KKA^y^ and 12 sex-matched C57BL/6J mice at the age of 8 weeks were purchased from the Chinese Academy of Medical Sciences (Beijing, China). The mice were housed under a controlled environment at 22 ± 1 °C and a 12-h light/dark cycle, with free access to food and water. Age-matched C57BL/6J mice were fed a normal chow diet, whereas the KKA^y^ mice were fed a high-fat diet. The Committee for Laboratory Animal Care and Use at the Shanghai University of Traditional Chinese Medicine approved all the experiments. An Institutional Review Committee for Animal Use approved the experimental protocol, and all procedures were in compliance with the National Institutes of Health Guide for Care and Use of Laboratory Animals (Publication No. 85-23, revised 1985).

### 3.3. Experimental Design

The design of the 84-day experiment was slightly modified compared with the previously published protocol [14]. Briefly, 12-week-old KKA^y^ mice were assigned to four treatment groups according to their fasting blood glucose values (first criterion) and initial body weights (second criterion). From the age of 12 weeks, the KKA^y^ mice were gavaged once daily with either distilled water, MDG-1 (150 or 300 mg/kg body weight/day) or RGZ (2 mg/kg body weight/day) for 12 weeks. At the same time, the lean mice were treated with distilled water. The fed blood glucose levels (tested at 8:30 AM) obtained from the tail veins of the mice were tested every two weeks using a One-Touch Basic Glucose Monitor (Lifescan, Milpitas, CA, USA). Body weight was also measured at the same time. At the mid-point and the end of the experimental period, food was withheld from the mice for 6 h at 9:00 AM, and blood samples were obtained from the orbital sinus to determine serum insulin levels.

At the end of the experiment, each group of mice was sacrificed by exsanguination under anesthesia at regular intervals after 6 h of fasting. The blood of all mice was then centrifuged and the serum was divided into aliquots and stored at −80 °C until analysis. After blood collection, both kidneys were removed, rinsed with a physiological saline solution and weighed. One kidney was fixed in 10% formalin solution for histological examination, while the other was snap frozen in liquid nitrogen and stored at −80 °C for protein isolation.

### 3.4. Biochemical Determinations

After the 12-week treatment period, blood was collected into tubes without anticoagulant from the retro-orbital sinus for clinical chemistry studies. It was allowed to clot, then centrifuged to obtain serum and was then snap frozen at −80 °C. A fully automated biochemical analyzer (7600-020, Hitachi, Tokyo, Japan) was used to measure the following parameters: Triglycerides (TG), blood urine nitrogen (BUN) and albumin. A rat/mouse enzyme-linked immunosorbent assay (ELISA) determined the serum insulin levels.

### 3.5. Histopathological Examination

The kidney tissues were immersed-fixed in 10% buffered formalin and embedded in paraffin for light microscopy studies (BX-51, Olympus, Tokyo, Japan). Sections of 3 µm thickness were stained with Periodic Acid-Shiff (PAS) or Masson’s trichrome stain to evaluate renal scarring and glomerular sclerosis (GS), and to better differentiate exudative lesions.

GS was assessed using a semi-quantitative analysis defined as the percentage of the mesangial matrix to document the degree of damage in a blinded fashion, as described previously [30]. In brief, a minimum of 30 glomeruli per mouse kidney were evaluated, with the mean used as the representative value for the mouse. The amount of mesangial ECM was identified by PAS-positive material in the mesangium. The glomeruli from the middle third of the renal cortex were selected for area measurements, using Image-Pro Plus 6.0 (Media Cybernetics Inc., Rockvill, MD, USA); extra care was taken to exclude the juxtamedullary glomeruli. The percentage of mesangial matrix occupying the selected glomerular tuft was then calculated.

The interstitial fibrosis score (as a percent) was assessed for each of the 10 microscopic fields on blue viewed at 400× magnification using Masson’s trichrome stain. A color image analyzer (Image-Pro Plus 6.0, Media Cybernetics Inc., Rockville, MD, USA) quantified the lesions. The fibrotic area was digitized and subjected to color threshold analysis. Scores from 10 non-overlapping fields per kidney were averaged as the final percentage of positive staining [31].

### 3.6. Western Blotting Assay

The cell lysates from the tissues were prepared in 1 mL lysis buffer on ice in 1.5 mL microtubes. They were then solubilized by continuous stirring for 30 min at 4 °C and centrifuged for 10–15 min at 14,000× *g*. The supernatants were collected and the protein concentrations were measured with a BCA protein assay reagent kit. The samples were stored at −80 °C until further analysis. The cell lysates were subjected to sodium dodecyl sulfate polyacrylamide gel electrophoresis (SDS-PAGE) and blotted onto a polyvinylidene difluoride membrane, followed by incubation with the anti-CTGF and anti-TGF-β_1_ primary antibodies. The enhanced-chemiluminescence (ECL) system was used to detect reactive proteins.

### 3.7. Data Analysis

Data were expressed as the mean ± standard deviation (SD). Statistical significance was determined using analysis of variance (ANOVA) with LED and Dunnett’s tests for post hoc comparisons at a 1% or 5% significance level of difference. The relationships between variables were determined by linear regression analysis after log transformation. Statistical analyses were performed using the SPSS 17.0 software package for Windows (SPSS, Chicago, IL, USA).

## 4. Conclusions

In conclusion, our results suggested that MDG-1 has a renoprotective effect via reduction of hyperglycemia, which attenuates the concentration of serum albumin and down-regulates the expression of TGF-β_1_ and CTGF in diabetic glomeruli, consequently decreasing ECM deposition in renal tissues. Hence, MDG-1 could be a promising drug candidate to prevent, or at least slow down, the progression of early diabetic nephropathy.
